# UAV Deployment Exercise for Mapping Purposes: Evaluation of Emergency Response Applications

**DOI:** 10.3390/s150715717

**Published:** 2015-07-02

**Authors:** Piero Boccardo, Filiberto Chiabrando, Furio Dutto, Fabio Giulio Tonolo, Andrea Lingua

**Affiliations:** 1 Politecnico di Torino—Interuniversity Department of Regional and Urban Studies and Planning (DIST), Viale Mattioli 39, 10125 Torino, Italy; E-Mail: piero.boccardo@polito.it; 2 Politecnico di Torino—Department of Environment, Land and Infrastructure Engineering (DIATI), C.so Duca degli Abruzzi 24, 10129 Torino, Italy; E-Mail: andrea.lingua@polito.it; 3 Città Metropolitana di Torino—Servizio Protezione Civile, Via Alberto Sordi 13, 10095 Grugliasco (TO), Italy; E-Mail: furio.dutto@cittametropolitana.torino.it; 4 Information Technology for Humanitarian Assistance, cooperation and Action (ITHACA), Via P.C. Boggio 31, 10138 Torino, Italy; E-Mail: fabio.giuliotonolo@ithaca.polito.it

**Keywords:** UAV, orthophoto, emergency response, photogrammetry, civil protection

## Abstract

Exploiting the decrease of costs related to UAV technology, the humanitarian community started piloting the use of similar systems in humanitarian crises several years ago in different application fields, *i.e.*, disaster mapping and information gathering, community capacity building, logistics and even transportation of goods. Part of the author’s group, composed of researchers in the field of applied geomatics, has been piloting the use of UAVs since 2006, with a specific focus on disaster management application. In the framework of such activities, a UAV deployment exercise was jointly organized with the Regional Civil Protection authority, mainly aimed at assessing the operational procedures to deploy UAVs for mapping purposes and the usability of the acquired data in an emergency response context. In the paper the technical features of the UAV platforms will be described, comparing the main advantages/disadvantages of fixed-wing *versus* rotor platforms. The main phases of the adopted operational procedure will be discussed and assessed especially in terms of time required to carry out each step, highlighting potential bottlenecks and in view of the national regulation framework, which is rapidly evolving. Different methodologies for the processing of the acquired data will be described and discussed, evaluating the fitness for emergency response applications.

## 1. Introduction

An unmanned aerial vehicle (UAV), commonly known as a drone and also referred to as an unpiloted aerial vehicle or a remotely-piloted aircraft (RPA) by the International Civil Aviation Organization (ICAO) is an aircraft without a human pilot aboard [1].

As highlighted in Office for the Coordination of Humanitarian Affairs (OCHA) [2], UAVs, previously mainly associated with military applications, are increasingly being adopted for civilian uses. UAVs are exploited in civilian (and also commercial) applications, like agriculture, surveying, video making and real estate. Exploiting the decrease of costs related to UAV technology, also the humanitarian community started piloting the use of similar systems in humanitarian crises several years ago in different application fields, *i.e.*, disaster mapping and information gathering, community capacity building, logistics and even transportation of goods.

Part of the author’s group, composed of researchers in the field of applied geomatics, has been piloting the use of UAVs since 2006 [3], with a specific focus on disaster management applications [4]. The present paper is therefore focused on the exploitation of UAVs in the emergency mapping domain, defined as “creation of maps, geo-information products and spatial analyses dedicated to providing situational awareness emergency management and immediate crisis information for response by means of extraction of reference (pre-event) and crisis (post-event) geographic information/data” [5]. Emergency mapping can be adopted in all of the phases of the emergency management cycle (*i.e.*, “the organization and management of resources and responsibilities for addressing all aspects of emergencies, in particular preparedness, response and initial recovery steps” [6], but the goal of the research is mainly to investigate potential applications supporting the immediate emergency response phase (“the provision of emergency services and public assistance during or immediately after a disaster in order to save lives, reduce health impacts, ensure public safety and meet the basic subsistence needs of the people affected” [6]).

As far as emergency mapping UAV applications are concerned, several organizations have already begun using the consolidated capability of UAVs to produce maps and provide high-resolution imagery as part of disaster response or disaster risk reduction programming. The main interest is in damage assessment information and in population count estimation [7]. 

Furthermore, taking the operational status of the current UAV technology into account, Politecnico di Torino decided to establish the Disaster Recovery Team (DIRECT [8]), a team of university students in the field of architecture and engineering with the goal to support disaster management activities through the application of geomatics techniques (3D surveying, remote sensing, mapping, WebGIS). In the framework of the DIRECT activities, a UAV deployment exercise was jointly organized with the Regional Civil Protection authority in July 2014 mainly aimed at assessing the effectiveness of the operational procedures in place to deploy UAVs for mapping purposes and the usability of the acquired data in an emergency response context.

The paper is structured in two main parts, focusing respectively on UAV deployment operational procedures (Section 3 and Section 4) and image post-processing (Section 5). In detail, Section 3 addresses the main phases of the adopted operational procedure, especially in terms of time required to carry out each step, highlighting potential bottlenecks, and in view of the national regulation framework, which is rapidly evolving. Furthermore the technical features of the UAVs platforms employed during the exercise will be described in Section 4. Different methodologies for the processing of the acquired data aimed at generating and distributing in near-real-time up-to-date orthoimages will be described and discussed in Section 5, with a specific focus on the positional accuracy and the fitness for emergency response applications.

## 2. UAV Deployment Exercise

In the framework of the DIRECT activities and in collaboration with the Provincial Civil Protection authority (Protezione Civile della Provincia di Torino), a UAV deployment exercise was organized in July 2014 with a two-fold aim: firstly, to test operationally UAV deployment procedures and secondly to assess the usability of the acquired high resolution imagery in an emergency response context. The present section will provide a detailed description of the deployment exercise setup.

**Figure 1:**
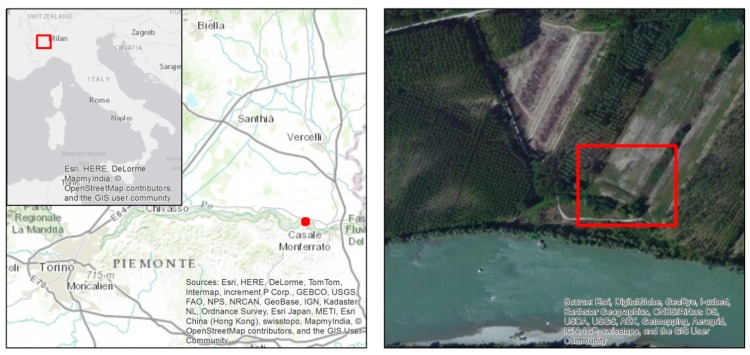
Deployment exercise location (**left**) and detail of the area to be mapped (**right**).

A specific area of interest (covering a surface of approximately 1.5 km^2^; Figure 1) to be mapped at different levels of detail was defined in advance, with the goal to simulate an area that could have been impacted by a crisis event, e.g., a plain flood.

Two different types of UAV platforms were planned to be tested, specifically a multi-rotor system and a fixed-wing airframe (described in Section 4), with the goal to evaluate the fitness for the purpose of similar platforms in an emergency context and possible specific limitations or advantages.

From an operation perspective and taking the immediate response phase into account, the main goal is to image and map the affected areas in the shortest time possible from the mobilization request, in order to provide to civil protection authorities updated imagery depicting the situation after the event. For this purpose, a specific list of operational phases was defined in advance, with the aim to evaluate the time required to carry out each step and, consequently, to identify potential bottlenecks to be further investigated and improved. The detailed description of the aforementioned steps and the outcomes of the evaluation is provided in Section 3.

As far as the orthoimage production is concerned, two different types of test were planned:

- firstly, a fast processing in the field (Section 5.2) exploiting as ground control points (GCP) pre-positioned markers to be quickly measured with low-cost devices and low positional accuracy (*i.e.*, mobile phones; Section 5.1);
- secondly, a rigorous processing (to be carried out off-line; Section 5.3) based on the same GCPs used in the previous test, but measured with a higher accuracy with topographical instrumentation, to be mainly used as a reference in the positional accuracy assessment phase (Section 5.4).

**Figure 2:**
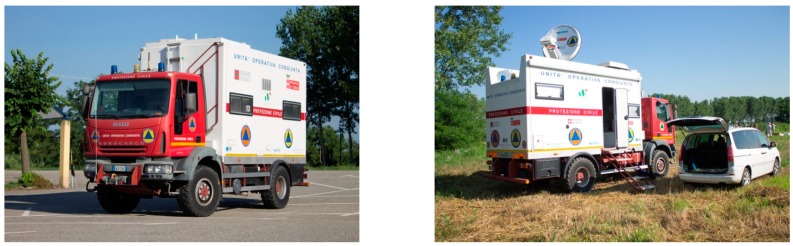
The Regional Civil Protection Mobile Unit, fully equipped with a complete “office” package (including web satellite connectivity): on its way to the test site (**left**) and deployed in the field (**right**).

Lastly, it was also planned to test the possibility to upload the processed image on a web mapping server directly from the field, exploiting the satellite connectivity offered by the fully-equipped mobile unit made available by the Regional Civil Protection authority (Figure 2).

## 3. UAV Deployment Operational Phases: Description and Discussion

As reported in the previous section, a complete list of the operational phases required to correctly carry out a UAV survey was defined in detail. Each phase and the related aim are described in this section: furthermore, the average time required to carry out the related tasks during the exercise is provided and discussed. 

Initial briefing: This phase follows a UAV service request to support emergency management activities (e.g., request to map damages to infrastructures in an industrial zone affected by a flood event). Preliminarily, it is necessary to get information on the location of the affected areas, mainly required to plan the deployment of the team and the instruments in the field and to identify an area suitable to be used as a coordination center in the field (allowing UAV’s taking-off and landing operations). Secondly, the requirements of the users requesting the service are analyzed, these being strictly connected to the technology to be adopted (e.g., mapping of a single building with rotor platforms *versus* mapping wide areas with fixed-wing airframes) and the technical details of the acquisitions (e.g., spatial/spectral resolution of the sensors installed on the UAV, average flight height, *etc.*). Lastly, a thorough assessment of the request is carried out, also taking local and/or international regulations into account, for a final decision of the feasibility of a UAV deployment.

This phase took about 30–45 min during the deployment exercise in the field, but it has to be considered that this was made possible mainly by the controlled environment of a simulated exercise. According to the authors’ experience, this phase could be one of the most time-consuming tasks, especially considering the specific planning activities and risk analyses to be carried out on a case by case basis according to the (evolving) Italian UAV regulation provided by Ente Nazionale Aviazione Civile (ENAC [9]).

Deployment of the team in the affected area: This phase is required to allow the mapping team (and the related hardware instrumentation, *i.e.*, UAV platforms, ground station, mobile devices for GCP collection, markers, *etc.*) to reach the selected operation base. 

The time required to carry out this operation is obviously strictly related to the organization of the institution managing the UAV deployment and the possible pre-positioning of UAV teams in areas at risk or allowing a complete local/regional/national coverage: the time span could therefore range from a few hours (as in the exercise case) to days.

Remotely-piloted aircraft system setup: This phase is aimed at correctly setting up the hardware and software required for the UAV mission and carrying out the flight operations, as well as the subsequent image downloading and related post-processing (if required). During the exercise, this preliminary phase took about 20 min.

Marker pre-positioning and survey: In order to grant an adequate positional accuracy of the processed orthophoto, it is required to identify on the acquired images features of interest whose coordinates with respect to a defined reference system are known with a suitable accuracy. Considering that most often in operational cases, large-scale maps (with a sufficient level of detail) are not available or the accuracy achievable exploiting global map services (e.g., Google Maps, OpenStreetMap) is not considered sufficient for the aim of the survey, it may be necessary to pre-position *ad hoc* markers that will be clearly visible in the acquired imagery. During the positioning operations, which should be carefully planned in the case of large areas to be surveyed to grant a homogeneous distribution of the markers and to speed up the process, the coordinates of the marker should be measured at the same time for efficiency purposes (see Section 5.1 for details).

The positioning and measuring of 16 markers during the exercise took approximately 1 h.

Flight plan setup: This task is crucial to plan the flight operations, and it is strictly related to the required technical features of the final map products, mainly in terms of spatial resolution and required overlapping percentages between images (to allow a proper photogrammetric 3D processing). UAV systems include *ad hoc* software to semi-automatically generated flight/acquisition plans to be uploaded onto the autopilot system. A skilled operator should be able to carry out this task in a few minutes: this was the case during the exercise.

UAV flight operations: This phase includes all of the tasks required to operate the UAV and to properly acquire the required data, including taking-off, landing and data downloading operations. The flight tests performed during the exercises took an average time of 15 min, ranging from 9 min (at a higher flight height) with rotor platforms to 17 min (covering a larger area) with fixed-wing platforms. It has to be noted that fixed-wing platforms are generally capable of covering larger areas in a shorter time than rotor platforms. It is also stressed the need to have redundant batteries (or even platforms) to optimize the mission time, limiting as much as possible the overall downtime.

Data processing (orthophoto generation): Once the raw images are downloaded, it is required to process the data in order to generate the required value-added products, including pre-processing tasks as data resampling or image selection (to limit the processing only to the minimum set of images actually required to cover the affected areas). The average time required to carry out this step during the exercise was about 2 h.

Data dissemination: This is the last phase of the activities, which is aimed to make the generated products (e.g., orthophoto) available to the users (which may be in remote locations, e.g., national civil protection headquarters). During the exercise, this step was carried out exploiting an *ad hoc* web map server, making the final orthoimage accessible as a standard OGC (Open Geospatial Consortium) WMS (Web Map Service). Image uploading and service setup required about 10 min; clearly, the actual time is strictly related to the data file size and the available upload bandwidth. 

It has to be highlighted that some of the aforementioned operations can be carried out in parallel in order to minimize the overall acquisition time, obviously depending on the number of people composing the team.

## 4. Employed UAV Platforms and Sensors

The UAV deployment exercise was planned to test two different platforms: a multi-rotor RPAS (Remotely Piloted Aircraft System) and a fixed-wing system.

### 4.1. Rotor Platform and Sensor

**Figure 3:**
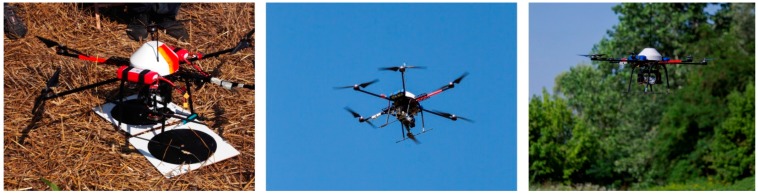
Hexakopter multi-rotor platform (**left**) and take-off and flying phases (**center** and **right**).

The first test was performed over an area of 400 m × 400 m using a Hexakopter by Mikrokopter (Figure 3).

The system (technical details are available at [10]) is composed of six motors and the electronic equipment required for both the remote control and the automatic flight (*i.e.*, one flight control adaptor card, one remote control, one navigation control, one GPS receiver, a three-axis magnetometer, one wireless connection kit) as well as one computer serving as the ground control station.

As far as the image acquisition sensor is concerned, the multi-rotor platform is equipped with a commercial off-the-shelf (COTS) Sony Nex 5 digital mirror less camera. The digital camera is mounted on a servo-assisted support that grants electronically-controlled rotations along two directions (the x- and h-axis) with the goal to acquire vertical imagery (lens axis pointing at nadir).

In Table 1, the main technical features of the employed multi-rotor platform are reported. 

**Table 1:** Main technical features of the employed multi-rotor platform.

Hardware	
Weight	2.5 kg
Payload	1 kg
Propulsion	Electric
Camera (focal length = 16 mm)	Sony Nex 5 (pixel size 5.22 µm)
**Operation**	
Maximum flight time	12–15 min
Nominal cruise speed	3–5 m/s
Ground Sampling distance at 100 m	0.032 m
Linear landing accuracy	Approximately 1 m

**Figure 4:**
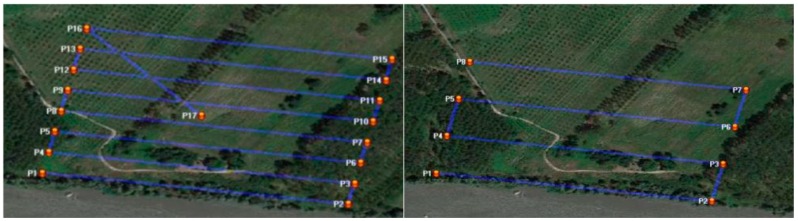
The multi-rotor RPAS planned flight plans: 70-m flight height above the ground (**left**) and 150-m flight height above the ground flight (**right**).

In order to have two different sets of test images and to simulate operations in the two volumes of space mentioned by the Italian RPAS regulation, two different flights were planned and carried out. One flight with a height of 70 m (the maximum height above the ground allowed by volume of space V70 according to the ENAC regulation) and a second flight performed at 150 m (the maximum height above the ground allowed by volume of space V150 according to the ENAC regulation). In the first case, the area was covered with eight stripes and 190 images characterized by a ground sample distance (GSD) of 0.022 m. The second flight allowed the coverage of the area with four stripes and 120 images with a GSD of 0.05 m. The longitudinal and lateral overlapping were respectively set at 80% and 30% in both cases. Using the Hexakopter, the flight plan is managed by the Mikrokopter tool OSD that connects the platform to the ground station. The tool is exploited to set all of the parameters of the flight plan, using as a reference map of the area one of the images made available by several on-line map servers (e.g., Google Maps, Bing Maps, *etc.*). The flight planning approach is the usual one based on the definition of waypoints (a reference point in the physical space used for the navigation, usually placed at the beginning and at the end of the stripes).The RPAS aims to follow the direction between the starting and ending points according to the defined azimuth. In Figure 4, two screen shots of the planned flight plans are shown. A good practice is also to insert the last waypoint close to the take-off and landing area (Figure 4 (left), waypoint P17), in order to simplify the manual landing operation. 

Using the Mikrokopter tool, several other parameters could be set, such as flight speed, elevation, shooting time, *etc*. According to the aim of the test, the time required to carry out the relevant phases of these flights was recorded. In the first case (70 m flight height), 10 min were required for the planning phase and 13 min for flying over the area. The second flight (150 m flight height) was planned in 5 min, and the images were acquired in 9 min. Finally, the images stored in the SD card were downloaded on a laptop (for the post-processing phase) in about 5 min.

### 4.2. Fixed-Wing Platform and Sensor

**Figure 5:**
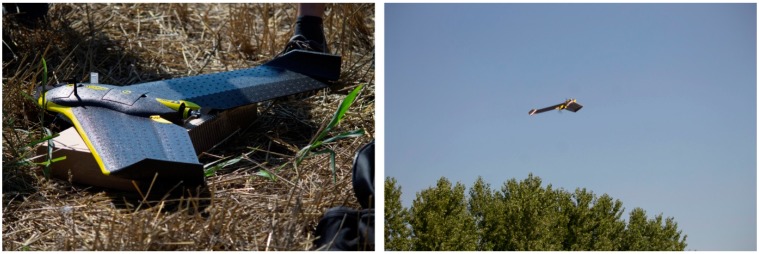
The eBee fixed-wing platform (**left**) and take-off phase (**right**).

A fixed-wing RPAS was also evaluated. The platform employed in the test was a consumer product of the SenseFly company (technical details are available at [11]), namely the eBee autonomous flying drone (Figure 5).

During the UAV deployment exercise, the system was used with an RGB Canon Ixus COTS camera to acquire visible imagery. Other sensors, tailored to the system, are also available, *i.e.*, a near-infrared camera (to acquire false color imagery), the multi-SPEC 4C camera that acquires multi-spectral images (green, red, red-edge and near-infrared) and the thermal sensor that produces grey scale images and video with high pixel density and thermal resolution.

The main technical features of the platform are reported in Table 2. 

**Table 2:** Main technical features of the employed fixed-wing RPAS.

Hardware	
Weight (camera included)	0.700 kg
Wingspan	96 cm
Propulsion	Electric
Camera (focal length = 4.3 mm)	16 Mp Ixus/ELPH (pixel size 1.33 µm)
**Operation**	
Maximum flight time	50 min
Nominal cruise speed	11–25 m/s
Ground Sampling distance at 100 m	0.031 m
Linear landing accuracy	Approximately 5 m

**Figure 6:**
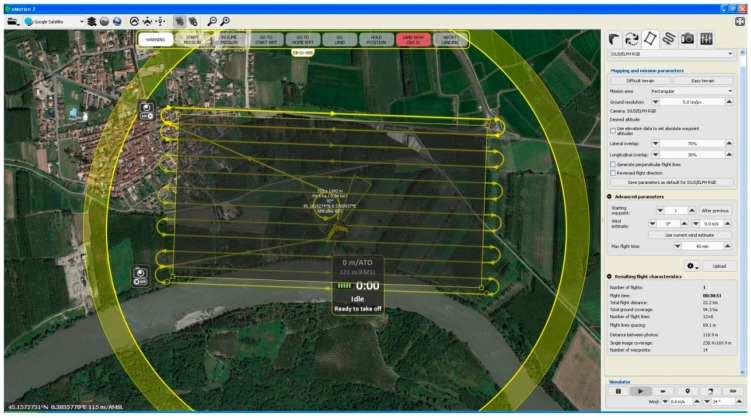
The fixed-wing RPAS flight plans: 150-m flight height above the ground.

The area selected for the test of the fixed-wing platform flight was approximately 1 km^2^. In order to have a GSD suitable for producing an orthophoto fitting a map scale of about 1:500, the flight height was set at 150 m. Sixteen stripes and 160 images were acquired with a 70% longitudinal and 30% lateral overlapping (Figure 6). The GSD (of the acquired images according to the aforementioned parameters was about 0.05 m. 

The eBee flight plan is managed through the eMotion software by SenseFly, which is based on an automatic photogrammetric approach. Using eMotion, the initial parameters to set up are the ones related to the area of interest, the GSD and the overlapping percentages between the images (lateral and longitudinal). Once these parameters are set, the software automatically calculates the number of stripes required to cover the area of interest, as well as the flight height. The second planning step is focused on take-off and landing settings. This step is obviously crucial, with RPAS automatically managing the landing phase, since an area without vertical obstacles in a certain range is absolutely necessary. The eBee performance during the landing phase is excellent, considering that (exploiting the onboard GNSS and ultrasound proximity sensors) the platform is able to land in a predefined area with an accuracy of about 5 m. 

As far as the time stamps are concerned, the flight planning was carried out in about 10 min, while the flight time was 17 min. The raw images’ download was carried out in about 5 min.

### 4.3. Summary of the Time Required for Each Flight Step

Table 3 summarizes the time required for flight planning, flight execution and image downloading in each performed tests.

**Table 3:** Time required for each flight step for the different test configurations.

Platform and Flight Height	Planning (min)	Flight Time (min)	Data Download (min)	Acquired Area (km^2^)
Microkopter (70 m)	10	13	5	0.15
Microkopter (150 m)	5	9	5	0.20
eBee (150 m)	10	17	5	1

## 5. Orthophoto Generation and Accuracy Assessment

One of the aims of the test was the generation of a cartographic product able to document from the geometric and thematic perspective the surveyed area. An orthophoto is a geospatial dataset meeting these requirements, since it is a cartographic product able to provide both geometrically-corrected information (with uniform scale and corrected distortions) and radiometric information. In order to perform the orthorectification process of a set of images (covering the surveyed area from different points of view) it is necessary to: (i) estimate the position and attitude of the images (external orientation parameters) in a defined reference system (cartographic or local); and (ii) have the elevation model of the area, generally extracted during the processing as a digital surface model (DSM).

The aforementioned process is based on the photogrammetric approach. Currently, a fast and cost-effective approach for the estimation of the interior and external orientation, the extraction of the DSM and the production of the orthophoto is based on the SfM (structure from motion [12]) methodology, implementing the usual photogrammetric workflow through computationally-efficient computer vision algorithms.

**Figure 7:**
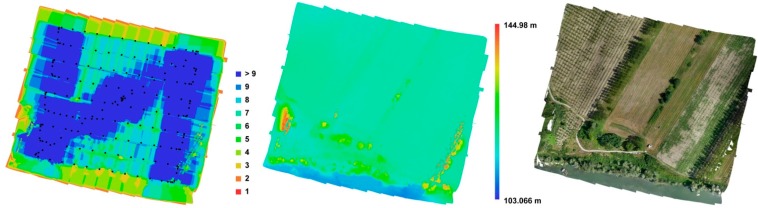
Oriented images with the number of overlaps (**left**), DSM (**center**) and orthoimagery (**right**).

In the framework of this test, Photoscan software (by Agisoft) was employed, which is one of the most popular tools used for 3D reconstruction purposes in the international community [13,14,15,16]. Being COTS software, very few algorithm details are available. The tie points extraction is performed using a modified SIFT (scale-invariant feature transform, [17]) approach. The external orientation (bundle block adjustment) is performed using the most common algorithm of the computer vision community, namely the Gauss–Markov approach [18]. The image matching seems to be realized using a semi global matching-like approach [19]. After the point cloud extraction step, as final products, a 3D model (Triangulated Irregular Network—TIN format), textures, a DSM and, eventually, the orthophoto are generated (Figure 7). 

In the following section, the strategy used for the realization of the orthophoto is reported. 

In order to evaluate different scenarios, the data processing steps were grouped according to the characteristic of the flights and the number of GCPs used for the bundle block adjustment.

Naturally, all of the processing steps were evaluated in terms of time (starting from the ground control point acquisition up to the web upload through a map server) and the positional accuracy of the realized cartographic product. 

### 5.1. GCP Acquisition

The correct workflow to generate a geospatial product with controlled positional accuracy requires the coordinates of several ground control points (GCPs) to be used in the georeferencing process (unless a direct georeferencing approach is adopted, as in one of the eBee-related tests) and for the accuracy evaluation. For this purpose, at least four GCPs are required, but a redundant number of points are obviously measured. Usually, a good compromise is ten points in a square kilometer [20,21].

**Figure 8:**
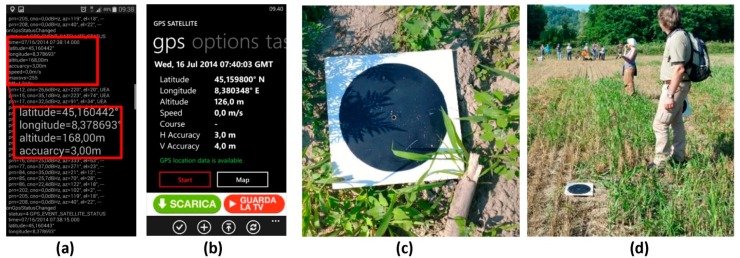
GPS coordinates measured by Samsung S4 with the GPS-GPX logger app (**a**), with the Nokia Lumia app (**b**); Details of the measured markers (**c**,**d**).

In certain conditions, it is not possible to easily identify natural features (to be used as GCP) on the images; therefore, it is necessary to pre-position *ad hoc* markers before the UAV flight. Those markers should homogeneously cover the whole area of interest and should be easily pin-pointed on the images. During the deployment exercise, 16 square wood panels (40 cm × 40 cm) with a black circle on a white background were use as markers (Figure 8c,d). 

The markers were then measured with two different approaches: firstly using a user-friendly device (e.g., a common GPS-equipped smartphone with a suitable app with the aim to test the results achievable with a user-friendly and low-cost technology) and then by means of a real-time kinematic (RTK) GPS survey, to have higher precision to be used as a reference in the accuracy assessment phase.

In the first case, during the test, the GCPs were measured using two different mobile applications available for Android (U-Center) and Windows (GPS-GPX logger), respectively installed on a Samsung Galaxy S5 and a Lumia 1020, involving two different teams (three persons each).

With the employed smartphones, the obtained precision according to the results reported in the employed apps was about 3 m, as expected with mobile GPS embedded sensors [22]. The GCP survey phase took about 45 min. Figure 8 shows a screenshot of one of the employed apps (a, b) and a detail of the marker (c, d).

Concerning the real-time kinematic (RTK) GPS survey, as expected with a GNSS RTK survey, the coordinates of the GCPs were measured with a horizontal accuracy of 0.02 m and a vertical accuracy of about 0.04 m. 

In this case, the survey was quite time consuming compared to the previous one: the points were surveyed in about 55 min with a Leica GNSS system 1200 using a virtual reference station (VRS) approach [23].

Table 4 summarizes the number of acquired points, the acquisition time and the mean accuracy of the two methods.

**Table 4:** Time required for ground control point (GCP) survey and average estimated accuracy. RTK, real-time kinematic.

Survey System	GCPs (No.)	Acquisition Time (min)	Average Planimetric Accuracy (m)	Average Elevation Accuracy (m)
Smart phone	16	45	3	3
GNSS-RTK	16	55	0.15	0.20

### 5.2. Orthophoto Generation: Fast Processing in the Field

The acquired images and GCPs coordinates were then exploited to generate an orthophoto with Photoscan. The software workflow is based on: photo alignment, dense cloud generation, georeferencing, mesh generation, texture generation and orthophoto export.

**Figure 9:**
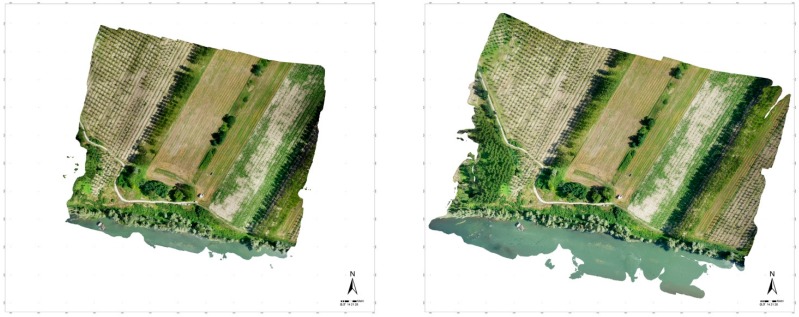
Orthophoto based on the images acquired with the multi-rotor UAV: 70-m flight height (**left**) and 150-m flight height (**right**).

To speed up all of the operations (the goal being processing in the field to limit the processing time as much as possible), the accuracy of each step was set at the lower level possible. The image processing steps were carried out on a laptop with the following technical features: Pentium i7 2.40 GHz, 16 GB RAM with an Nvidia GeForce GTX 670 2 GB. The data acquired during the two multi-rotor flights (70-m and 150-m flight height) were processed using the GCPs’ coordinates with lower accuracy (the ones being quickly available in the field). Figure 9 shows the output orthophoto. 

**Figure 10:**
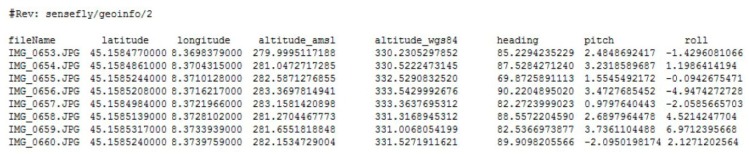
Example of the external orientation parameters as measured by the eBee platform, required for the direct georeferencing approach.

The eBee data were processed with a direct georeferencing approach exploiting the GPS/IMU position/attitude measurements as initial approximate values [24]. The sensors installed on the eBee platform are a GPS chip (U-Blox chipset), which provides a position based on C/A (Coarse/Acquisition) code at 1 Hz (no raw code data is recorded), and an attitude sensor, which provides the three attitude angles (roll, pitch and heading). In order to import those parameters in Photoscan, the export option of the eMotion software was exploited to get an ASCII file with the coordinates (latitude, longitude, altitude) and attitude (heading, pitch and roll) of each acquired image (Figure 10). 

**Figure 11:**
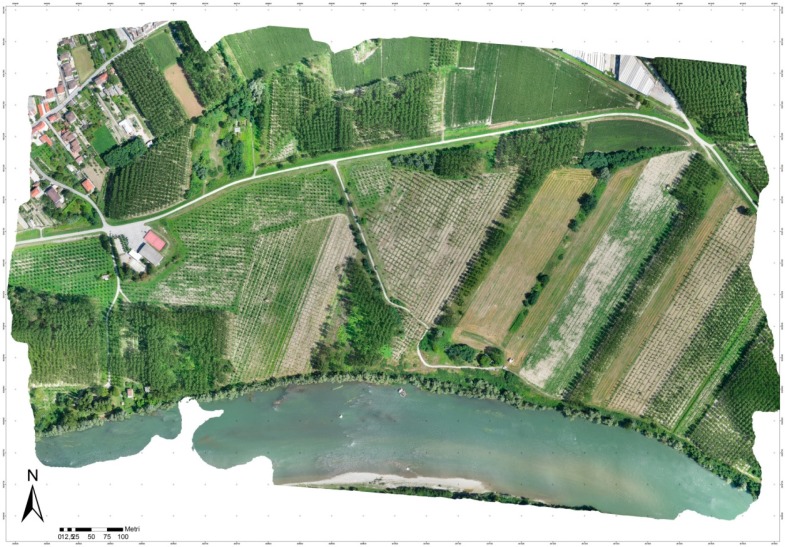
Orthophoto based on the images acquired with the fixed-wing UAV (direct georeferencing approach).

All of the processing steps carried out with the images acquired by the multi-rotor platform were applied also for the eBee data, obviously excluding the GCP step. Figure 11 shows the final orthophoto.

For comparison purposes, the same data were re-processed also using as input the GCPs measured with smartphones. Table 5 summarizes the main settings of the different data processing in the field. 

**Table 5:** Main settings of the image processing step in the field.

Platform	Flight Height (m)	Employed GCPs Measured with Smartphones (No.)	Processed Images (No.)	GSD (m)	Processing Time (h:min:s)
Multi-rotor	70	6	190	0.044	1:43:00
Multi-rotor	150	10	120	0.091	1:40:00
eBee	150	N/A	163	0.040	1:50:00
eBee	150	10	163	0.040	1:55:00

In conclusion, the test proved multi-rotor platforms to be more flexible, allowing one to carry out the take-off and landing operations in smaller areas even with vertical obstacles. Furthermore, their flight height can be lower than the one required by a fixed-wing platform, providing images with higher GSD and more flexibility in setting the required photogrammetric parameters (e.g., stereo pair base to optimize the estimated vertical accuracy). These platforms are usually the first choice for surveying small areas or isolated buildings.

On the other hand, if large areas need to be mapped, the preferred approach is based on fixed-wing UAVs, allowing speeding up of the acquisition process (by flying at a 150-m height, it is possible to cover 1 km^2^ in about 15 min). The main disadvantage of these platforms is the need for relatively large landing areas (at least 50 m long) without vertical obstacles to allow the platform to correctly approach the landing point (although the eBee platform has also a circular landing mode to cope with this issue).

### 5.3. Orthophoto Generation: Rigorous Off-Line Processing 

In order to evaluate the possible differences in terms of accuracy and processing time between a fast processing approach (GCPs measured by smartphones and low accuracy processing setting, standard performance laptop) and a more rigorous approach (GCPs measured by GNSS RTK and medium/high accuracy processing setting, high performance desktop computer), all of the acquired data were re-processed in the Geomatics Laboratory of the Politecnico di Torino with a higher performance computer (Pentium i7 3.70 GHz, 16 GB RAM with two Nvidia GeForce GTX 7502 GB).

The Photoscan workflow was therefore set to exploit medium/high accuracy settings during the processing; specifically, a medium accuracy value was set for the tie point extraction step and a high accuracy value for the mesh generation phase. The time required for processing was shorter than the time required in the field, despite the more rigorous setting, thanks to the higher performance of the adopted desktop computer; on the other hand, the final results in terms of radiometric quality were comparable to the outputs generated in the field, demonstrating that even a fast processing in the field fulfils the emergency response requirements.

As expected, the GCPs measured with the RTK survey allowed improving of the positional accuracy of the orthophoto (the details of the accuracy assessment phase are provided in Section 5.4). 

Table 6 summarizes the main settings of the different data off-line processing. 

**Table 6:** Main settings of the off-line image processing step.

Platform	Flight Height (m)	Employed GCPs Measured with GNSS RTK (No.)	Processed Images (No.)	GSD (m)	Processing Time (h:min:s)
Multi-rotor	70	6	190	0.044	1:30:00
Multi-rotor	150	10	120	0.091	1:25:00
eBee	150	N/A	163	0.040	1:40:00
eBee	150	10	163	0.040	1:50:00

### 5.4. Accuracy Assessment Results

According to the typical geomatics researcher mind-set, all of the products were compared in order to assess the geometric accuracy, with the final goal to estimate the possible nominal map scale of the orthophoto (according to the Italian national mapping standards).

The accuracy was evaluated using as check points (CP) the most accurate surveyed coordinates (by means of a GNSS RTK survey). For the orthophoto based on GCPs measured by smartphones and on the direct georeferencing approach, all 16 points measured by the GNSS RTK survey were used as CP. For the orthophoto based on GCPs measured by the GNSS RTK survey, the accuracy evaluation was performed using as CP six points not employed in the photogrammetric process. 

Table 7 summarizes the average positional accuracy and the estimated nominal map scale of the orthophoto products.

**Table 7:** Results of the accuracy assessment phase of the orthophoto products.

Product ID	Platform	Flight Height (m)	Employed Strategy	Easting Average Accuracy (m)	Northing Average Accuracy (m)	Elevation Average Accuracy (m)	Nominal Scale (According to the Planimetric Error)
1	Multi-rotor	70	Smartphone GCPs	1.24	1.03	2.65	1:5000
2	Multi-rotor	70	RTK GCPs	0.06	0.03	0.04	1:500
3	Multi-rotor	150	Smartphone GCPs	1.10	0.78	6.45	1:5000
4	Multi-rotor	150	RTK GCPs	0.10	0. 25	0.36	1:1000
5	eBee	150	Direct geo	1.48	1.55	12.01	1:10,000
6	eBee	150	RTK GCPs	0.08	0.10	0.48	1:1000

According to the results summarized in Table 7, it is clear that the usage of GCPs measured by traditional GNSS survey techniques leads to higher positional accuracy that fits very large nominal map scales (Products 2, 4 and 6). On the other hand, it is also evident that the accuracies of orthophotos based on smartphone-measured GCPs (Products 1, 3), having displacements lower than 1.5 m, fit medium nominal scales that are indeed suitable for emergency mapping purposes. The more interesting consideration is the fact that the same level of accuracy can be obtained with a direct georeferencing approach (Product 6), without any need to measure GCPs in the fields, therefore reducing the overall time required to get usable post-event products and allowing technicians to carry out surveys also in areas with limited accessibility (where a proper GCP survey would not be feasible). It has to be highlighted that the direct georeferencing approach could be further improved using a more accurate GNSS sensor, such as the one embedded in the recently released RTK version of the eBee platform: the first tests highlight that centimeter accuracy can be obtained without any control points [25].

## 6. Example of Value-Added Application in the Emergency Mapping Domain

An up-to-date, very high resolution orthophoto is one of the main data sources exploited for emergency mapping activities, with the main goal to identify the most affected areas and to assess the damages to the main infrastructures, providing value-added products to end users. Typical value-added products are in the form of cartographic products, and generally, the underlying datasets are also provided to end users as vector layers.

Most often, the aforementioned input imagery is acquired from satellite platforms, due to the possibility to trigger a wide range of satellite sensors (both optical and SAR) with the goal to get usable images in the shortest time frame possible. Nevertheless, in some circumstances, satellite platforms may not be able to provide adequate imagery for several reasons: type of post-event analysis requiring a spatial resolution larger than the one offered by satellite sensors (*i.e.*, 0.3 m as of May 2015), persistency of cloud coverage and need for optical imagery, illumination conditions of the affected areas at the time of satellite passage (at very high latitudes), possible acquisition conflicts leading to repeated acquisition cancellations, orography of the affected areas leading to heavy geometrical distortions. In those cases, aerial imagery acquired by UAV platforms is a possible alternative data source to be used for the post-event analysis. Generally, UAV imagery is also considered an *in situ* dataset that can complement satellite-based analysis: the European Commission is already assessing the potential role of UAVs, using the collected imagery “as an alternative and/or complementary source of post-event imagery in emergency situations and in a rapid response and mapping context” [26].

When very high resolution optical imagery is available, the typical workflow to assess the damages to infrastructure is based on computer-aided photo interpretation (CAPI) techniques carried out by skilled image interpreters [27], which certainly benefits from the typical very high spatial resolution of UAV imagery.

**Figure 12:**
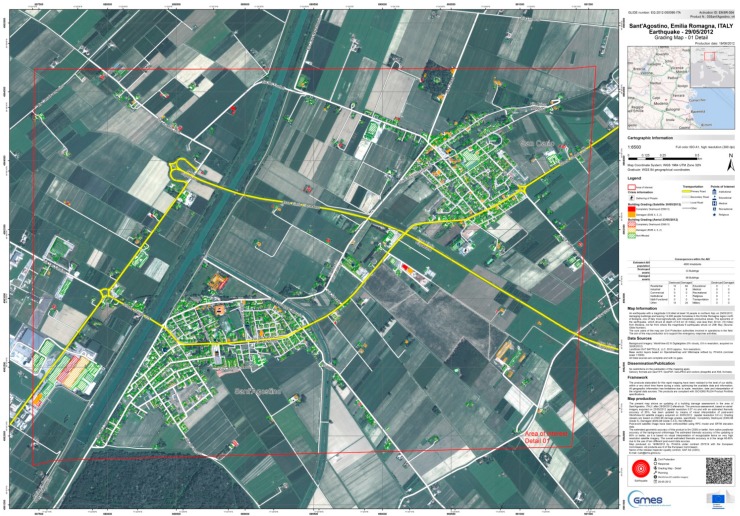
Example of post-event damage assessment map based on both aerial and satellite imagery (Copernicus Emergency Management Service–Mapping [26]).

Figure 12 shows an example of a value-added map produced during the response phase to an earthquake that struck Northern Italy on May 2012 (in the framework of the European Commission Copernicus Emergency Management Service Mapping) assessing damages to buildings on the basis of very high resolution optical imagery acquired from aerial and satellite platforms.

## 7. Discussion and Conclusions

As described in Section 2, the UAV deployment exercise had a two-fold aim: to operationally test UAV deployment procedures with a focus on the timeliness of the service and, secondly, to assess the usability of the acquired high resolution imagery in an emergency response context, especially in terms of spatial accuracy.

As far as the UAV deployment procedures are concerned, the evaluation of the time required to carry out each operational task highlighted that the more demanding steps are the ones required to initially assess the request and to deploy the team in the field. The team deployment operation can reasonably last from a few hours to days, especially considering possible accessibility issues most often characterizing disaster-affected areas. Once in the field, each technical procedure related to the flight survey and the image processing takes from a few minutes (e.g., flight plan planning) to a couple of hours maximum (e.g., marker GPS survey and image processing). The outcomes of the tests clearly demonstrate that an aerial survey of the affected areas (limited to a few square kilometers) can be technically carried out in a time frame fulfilling emergency management requirements (*i.e.*, to get up-to-date post-event information in the shortest time possible). The main bottleneck from the operational procedure perspective is related to the need to deploy all of the required instrumentation in the affected areas: in the framework of an operational service, the authors strongly believe that only pre-positioned stand-by UAV teams covering specific geographical areas may grant the possibility to reach the target area in a reasonable and limited timeframe (a few hours possibly). In the case of international operations, the time required to get valid visas (if necessary) has also to be taken into account. Furthermore, the existing (and rapidly evolving) national (and international) regulations should be thoroughly evaluated and addressed well in advance, to be sure that all the regulation-related tasks (e.g., special flight permission requests or the request to issue a NoTAM, Notice To AirMen) can be carried out quickly and correctly. A discussion with the relevant aviation authorities focused on the specific domain of RPAS for emergency management purposes should be also encouraged.

Adverse weather conditions (scarce visibility, heavy rains, strong winds) can also play an important role in possible delays of flight operations once in the field, due to the platforms’ technical limitations (although waterproof platforms exist, the capability to withstand strong winds is limited to certain air speed thresholds).

Concerning the extent of the area to be surveyed, multi-rotor platforms are usually the first choice when small areas or isolated buildings must be surveyed, therefore fitting the requirements of emergencies like industrial accidents (including potential needs for indoor flights in case of search and rescue applications [28]) and landslides.

If larger areas need to be mapped, which is generally the case in disasters like floods and wildfires, the preferred approach is based on fixed-wing UAVs, allowing speeding up of the acquisition process (at a 150-m height, 1 km^2^ can be covered in about 15 min). The use of UAV fleets, covering in parallel different portions of the affected area, could be planned in case of events that involve vast regions as an alternative to a typical mission based on several subsequent flights of the same platform. Furthermore, it has to be highlighted that according to the authors’ experience, the use of a UAV-based approach fits the need to get detailed information over the most impacted areas: the overview of the situation over the whole affected area is generally based on satellite imagery. Concerning the image processing phase, the number of images to be processed and the need for almost completely automated algorithms lead to time-consuming operations to get a usable orthophoto (*i.e.*, a couple of hours of processing in the field to cover about 1 km^2^). The adoption of proper tools aimed at preliminary extracting the minimum number of images among the collected ones (downsampling them to the spatial resolution actually required to allow a proper post-event analysis) is therefore encouraged.

Rigorous positioning accuracy assessment tests (Section 5.4) clearly demonstrate that even using a direct georeferencing approach (*i.e.*, no need for measured GCP), it is possible to obtain horizontal accuracies of about 1.5 m. Similar values are obviously suitable for the immediate response phase, allowing the responders to uniquely identify the affected features and to assess their damage grade in the post-event imagery.

The availability of the satellite connectivity offered by the Regional Civil Protection Mobile Unit allowed the team to test also the upload of the processed image on a web mapping server directly from the field. The test was indeed successful, and it demonstrated that the data can be made available in a few minutes (the actual time is strictly related to the available bandwidth and the size of the imagery) to the broader humanitarian community, e.g., to crowd mapping volunteers, which can definitively speed up the post-event analysis (*i.e.*, identification of the affected areas and, when possible, the assessment of damages to main infrastructures).
